# A League of Public Health Nurses

**Published:** 1919-03-15

**Authors:** 


### A LEAGUE OF PUBLIC HEALTH NURSES.

THE new Association known as the League of Trained Nurses Engaged in Public Health Work held its initial public meeting at 11 Chandos Street, W., on Saturday under the presidency of Her Royal Highness Princess Christian. Briefly put, the objects of the new body were stated to be to promote high standards of qualification for those nurses entrusted with public health work, and to secure standards of remuneration in line with the qualifications and responsibilities. Applicants for membership must hold a certificate of three years' general training, and be engaged in some branch of public health work or hold a recognised certificate in sanitary science.

Princess Christian's interest in nurses in general was expressed in the graceful sentence, "It is always a pleasure to me to meet nurses, and to know that they regard me as a friend; I can assure them that they have got no better friend." As to the particular purpose of the gathering, Her Royal Highness said such a conference could not fail to be useful at this time, when so many problems in connection with health work have to be faced. Subsequent speakers included the Rev. R. C. Gillie, Dr. Margaret Rooke, Miss Le Geyt, Miss Jessie Holmes, Miss Margaret Alderman, and Miss Wise.

For the most part the speeches were non-controversial, but Miss Le Geyt permitted herself the luxury of saying there were two Registration Bills before Parliament, and for her part she stood for the Central Committee's Bill, which was "an honest measure" [?] and had "nothing to do with such things as supplementary Registers." Two resolutions were passed. The first stated that "in the interests of the public and trained nurses alike it is expedient that a Bill should be introduced into the House of Commons at the earliest possible moment to provide for the registration by the State of fully-trained and qualified nurses; that such shall be free from bias towards any one organisation or another; and, further, that the Bill should provide for a one-portal system of three years' hospital training to qualify nurses for admission to the Register." The second resolution laid it down that "if a State Nursing Service be established under the Ministry of Health there shall be appointed an Advisory Council of trained nurses to advise on all questions relating to nurses."

